# The genome sequence of the Locust Fly, *Stomorhina lunata *(Fabricius, 1805)

**DOI:** 10.12688/wellcomeopenres.19532.1

**Published:** 2023-08-02

**Authors:** Ryan Mitchell, Olga Sivell, Natural History Museum Genome Acquisition Lab, Darwin Tree of Life Barcoding collective, Wellcome Sanger Institute Tree of Life programme, Wellcome Sanger Institute Scientific Operations: DNA Pipelines collective, Tree of Life Core Informatics collective, Darwin Tree of Life Consortium

**Affiliations:** 1 Natural History Museum, London, England, UK

**Keywords:** Stomorhina lunata, Locust Fly, genome sequence, chromosomal, Diptera

## Abstract

We present a genome assembly from an individual female *Stomorhina lunata* (the Locust Fly; Arthropoda; Insecta; Diptera; Rhiniidae). The genome sequence is 728.1 megabases in span. Most of the assembly is scaffolded into 6 chromosomal pseudomolecules, including the X sex chromosome. The mitochondrial genome has also been assembled and is 16.49 kilobases in length. Gene annotation of this assembly on Ensembl identified 18,358 protein coding genes.

## Species taxonomy

Eukaryota; Metazoa; Eumetazoa; Bilateria; Protostomia; Ecdysozoa; Panarthropoda; Arthropoda; Mandibulata; Pancrustacea; Hexapoda; Insecta; Dicondylia; Pterygota; Neoptera; Endopterygota; Diptera; Brachycera; Muscomorpha; Eremoneura; Cyclorrhapha; Schizophora; Calyptratae; Oestroidea; Rhiniidae; *Stomorhina*; *Stomorhina lunata* (Fabricius, 1805) (NCBI:txid1606781).

## Background

*Stomorhina lunata* (Fabricius, 1805) is a species of fly from the family Calliphoridae (blow flies), subfamily Rhiniinae. Rhiniinae used to be considered as a separate family ( Kutty *et al.*, 2010), however, recently it has been reclassified as a subfamily and placed within Calliphoridae ( Yan *et al.*, 2021). This classification has been accepted in Britain ( Chandler, 2023).

*Stomorhina lunata* can be easily identified in the field due to its unique appearance. The thorax has three black undusted stripes, and the abdomen has yellow (male) or greyish-yellow (female) markings. The lower part of the face is shining black and strongly protruding as in some species of Syrphidae. Parts of the gena, postgena, occiput, anepisternum and katepisternum are yellow or white, with dense pale hairs. The live flies have stripy eyes, a feature that disappears in dead specimens ( Draber-Mońko, 2004; Rognes, 1991; Sivell, 2021). Body length 5–9 mm ( Rognes, 1991).

*Stomorhina lunata* is oviparous. In favourable conditions the whole life cycle takes a month ( Draber-Mońko, 2004). The larvae are predators of locust eggs, they have also been found developing in the nests of termites ( Cuthbertson, 1933; Hall, 1948; Séguy, 1928) and *Annoma* (= *Dorylus*) ants ( Peris, 1956); *S. lunata* was one of three *Stormorhina* species collected as adults near nests of *Myrmica aimonissabaudiae* Menozzi, 1939 ants that were parasitised by *Stormorhina* larvae, although the identity of the larvae is unknown ( Bharti & Bharti, 2016). Greathead (1962) also observed them ovipositing on hen’s egg yolk and crushed locusts. The reported host species include the Moroccan Locust *Dociostaurus maroccanus* (Thunberg, 1815), Desert Locust *Schistocerca gregaria* (Forskål, 1775) (= *Schistocerca peregrina*), Migratory Locust *Locusta migratoria* (Linnaeus, 1758) and South African Brown Locust *Locustana pardalina* (Walker, 1870) ( Bharti & Bharti, 2016; Rognes, 1991). Adult *S. lunata* are often encountered on flowers, feeding on nectar and pollen ( Sivell, 2021; Wilkinson, 2022). The egg, larvae and pupa of *S. lunata* have been described by Cuthbertson (1933), Jannone (1947) and Hall (1948).

*Stomorhina lunata* is widely distributed in the Palaearctic, Afrotropical and Oriental Regions, and in Bermuda in the Nearctic Region ( Rognes, 1991; Thomas-Cabianca *et al.*, 2023). In Britain it is frequently seen during summer and early autumn. It is widely distributed in southern and central Britain, with a few recent records from Scotland ( Rhodes & MacDonald, 2018; Sivell, 2021; Wilkinson, 2022). The flight season is from July to October, with occasional records from June, November, and December ( Calliphoridae and Polleniidae Recording Scheme, 2023; Sivell, 2021). This species used to be considered an occasional vagrant in Britain ( Colyer, 1947; Dear, 1981; D’Assis-Fonseca, 1947; Van Emden, 1954; Wainwright, 1949), however in the past twenty years it has become more common and widely distributed and is now likely breeding locally ( Calliphoridae and Polleniidae Recording Scheme, 2023; Sivell, 2021; Wilkinson, 2022).

The high-quality genome of *Stomorhina lunata* was sequenced as part of the Darwin Tree of Life Project, a collaborative effort to sequence all named eukaryotic species in the Atlantic Archipelago of Britain and Ireland. Here we present a chromosomally complete genome sequence for *Stomorhina lunata*, based on a female from Hartslock Nature Reserve, England.

## Genome sequence report

**Figure 1.**
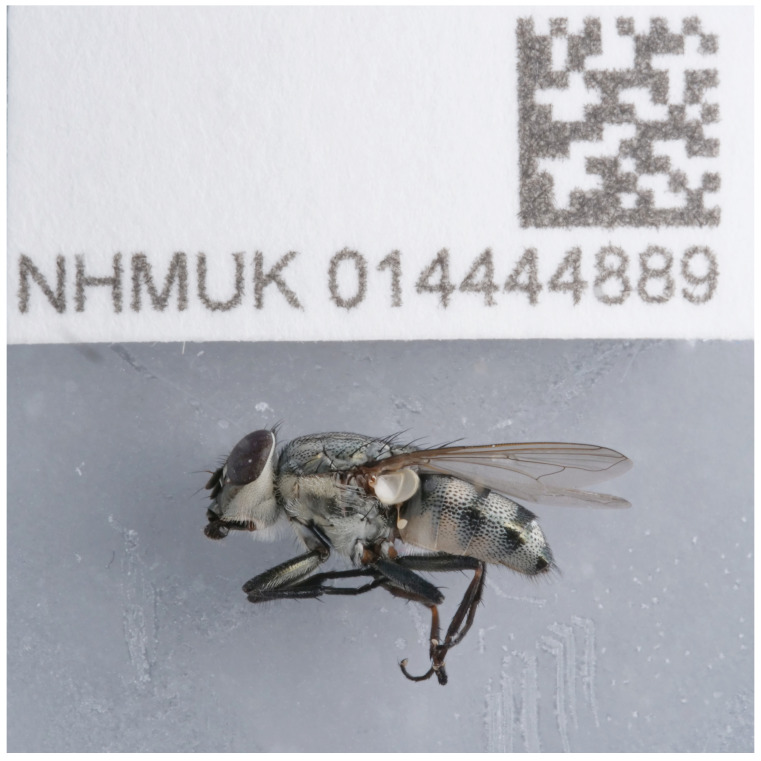
Photograph of the *Stomorhina lunata* (idStoLuna1) specimen used for genome sequencing.

The genome was sequenced from one female *Stomorhina lunata* ( Figure 1) collected from Hartslock Reserve, Oxfordshire, UK (51.51, –1.11). A total of 42-fold coverage in Pacific Biosciences single-molecule HiFi long reads was generated. Primary assembly contigs were scaffolded with chromosome conformation Hi-C data. Manual assembly curation corrected 155 missing joins or mis-joins and removed 24 haplotypic duplications, reducing the assembly length by 0.84% and the scaffold number by 38.51%, and increasing the scaffold N50 by 0.69%.

**Figure 2.**
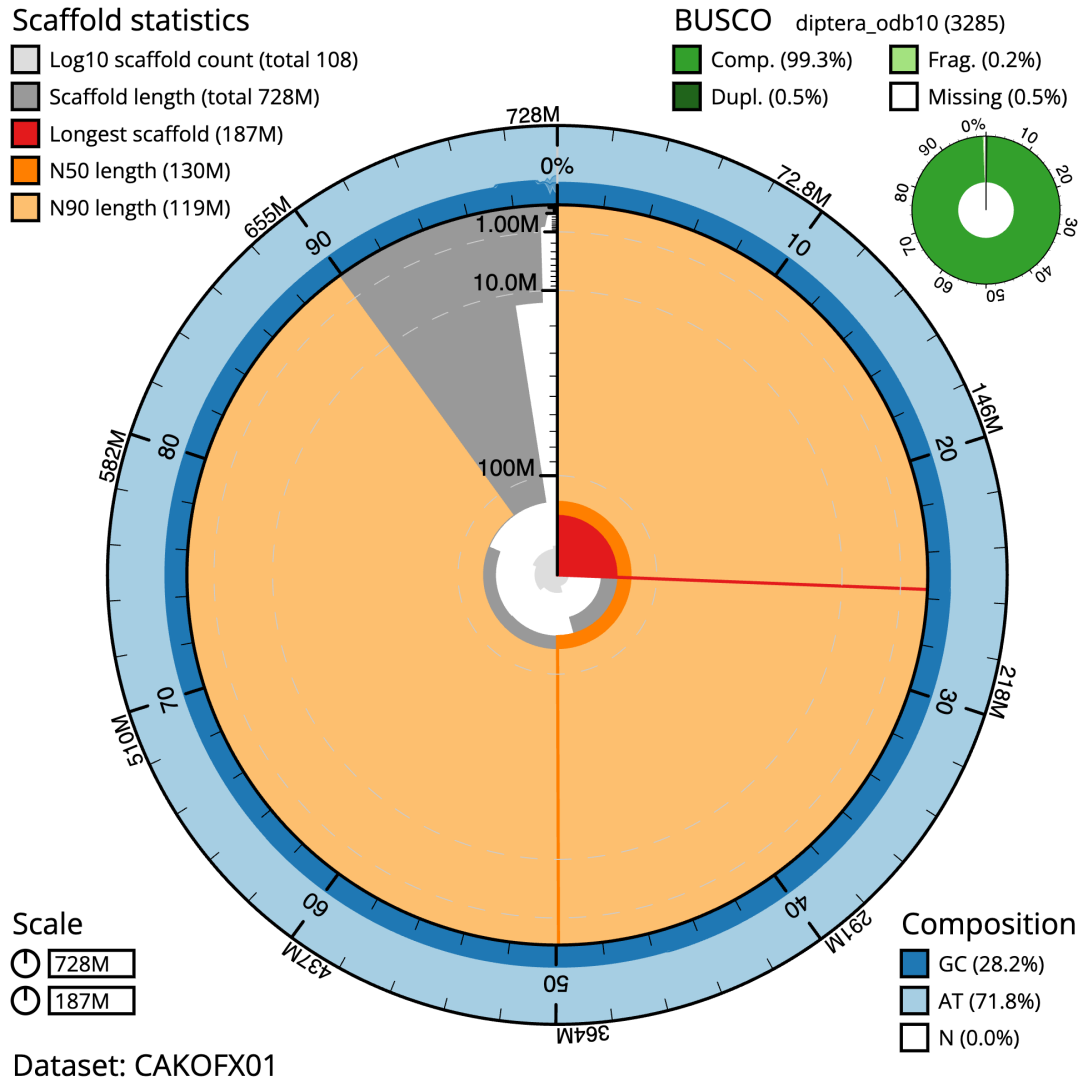
Genome assembly of *Stomorhina lunata*, idStoLuna1.1: metrics. The BlobToolKit Snailplot shows N50 metrics and BUSCO gene completeness. The main plot is divided into 1,000 size-ordered bins around the circumference with each bin representing 0.1% of the 728,100,380 bp assembly. The distribution of scaffold lengths is shown in dark grey with the plot radius scaled to the longest scaffold present in the assembly (186,698,790 bp, shown in red). Orange and pale-orange arcs show the N50 and N90 scaffold lengths (129,649,560 and 119,364,945 bp), respectively. The pale grey spiral shows the cumulative scaffold count on a log scale with white scale lines showing successive orders of magnitude. The blue and pale-blue area around the outside of the plot shows the distribution of GC, AT and N percentages in the same bins as the inner plot. A summary of complete, fragmented, duplicated and missing BUSCO genes in the diptera_odb10 set is shown in the top right. An interactive version of this figure is available at https://blobtoolkit.genomehubs.org/view/idStoLuna1.1/dataset/CAKOFX01/snail.

The final assembly has a total length of 728.1 Mb in 107 sequence scaffolds with a scaffold N50 of 129.6 Mb ( Table 1). Most (99.51%) of the assembly sequence was assigned to 6 chromosomal-level scaffolds, representing 5 autosomes and the X sex chromosome. Chromosome-scale scaffolds confirmed by the Hi-C data are named in order of size ( Figure 2– Figure 5; Table 2). While not fully phased, the assembly deposited is of one haplotype. Contigs corresponding to the second haplotype have also been deposited. The mitochondrial genome was also assembled and can be found as a contig within the multifasta file of the genome submission.

**Table 1.** Genome data for *Stomorhina lunata*, idStoLuna1.1.

Project accession data		
Assembly identifier	idStoLuna1.1	
Species	*Stomorhina lunata*	
Specimen	idStoLuna1	
NCBI taxonomy ID	1606781	
BioProject	PRJEB50881	
BioSample ID	SAMEA7849406	
Isolate information	idStoLuna1; thorax (DNA sequencing), head (HiC scaffolding), abdomen (RNA sequencing)	
Assembly metrics *		*Benchmark*
Consensus quality (QV)	58	*≥ 50*
*k*-mer completeness	99.99%	*≥ 95%*
BUSCO **	C:99.3%[S:98.8%,D:0.5%], F:0.2%,M:0.5%,n:3,285	*C ≥ 95%*
Percentage of assembly mapped to chromosomes	99.51%	*≥ 95%*
Sex chromosomes	X chromosome	*localised homologous pairs*
Organelles	Mitochondrial genome assembled	*complete single alleles*
Raw data accessions		
PacificBiosciences SEQUEL II	ERR8705865, ERR8705866	
Hi-C Illumina	ERR8702789	
PolyA RNA-Seq Illumina	ERR10123679	
Genome assembly		
Assembly accession	GCA_933228675.1	
*Accession of alternate * *haplotype*	GCA_933228635.1	
Span (Mb)	728.1	
Number of contigs	759	
Contig N50 length (Mb)	2.0	
Number of scaffolds	107	
Scaffold N50 length (Mb)	129.6	
Longest scaffold (Mb)	186.7	
Genome annotation		
Number of protein-coding genes	11,857	
Number of non-coding genes	1,492	
Number of gene transcripts	18,358	

* Assembly metric benchmarks are adapted from column VGP-2020 of “Table 1: Proposed standards and metrics for defining genome assembly quality” from ( Rhie *et al.*, 2021). ** BUSCO scores based on the diptera_odb10 BUSCO set using v5.3.2. C = complete [S = single copy, D = duplicated], F = fragmented, M = missing, n = number of orthologues in comparison. A full set of BUSCO scores is available at https://blobtoolkit.genomehubs.org/view/idStoLuna1.1/dataset/CAKOFX01/busco.

**Figure 3.**
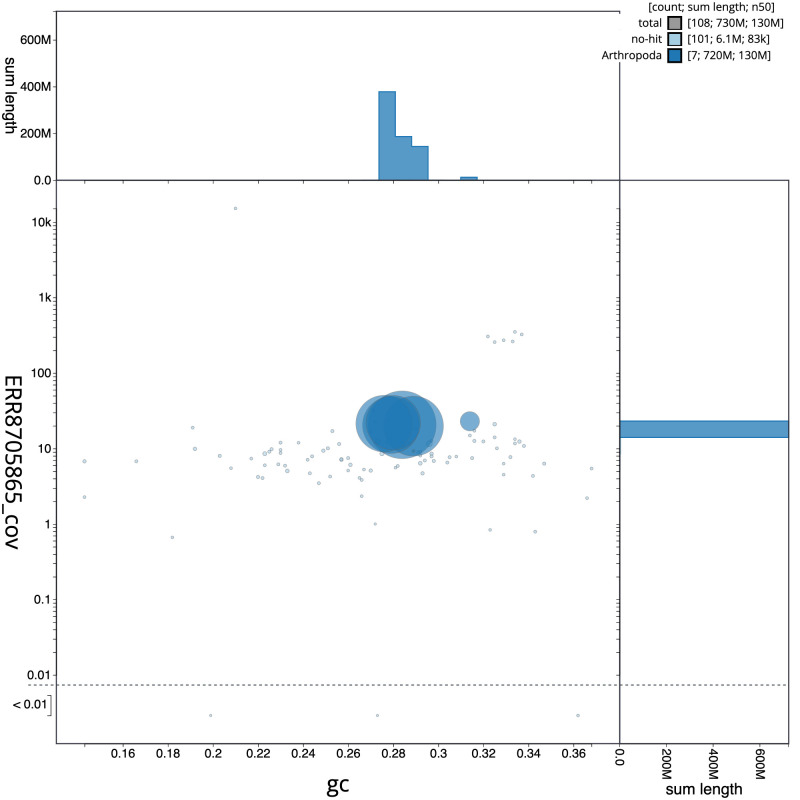
Genome assembly of *Stomorhina lunata*, idStoLuna1.1: BlobToolKit GC-coverage plot. Scaffolds are coloured by phylum. Circles are sized in proportion to scaffold length. Histograms show the distribution of scaffold length sum along each axis. An interactive version of this figure is available at https://blobtoolkit.genomehubs.org/view/idStoLuna1.1/dataset/CAKOFX01/blob.

**Figure 4.**
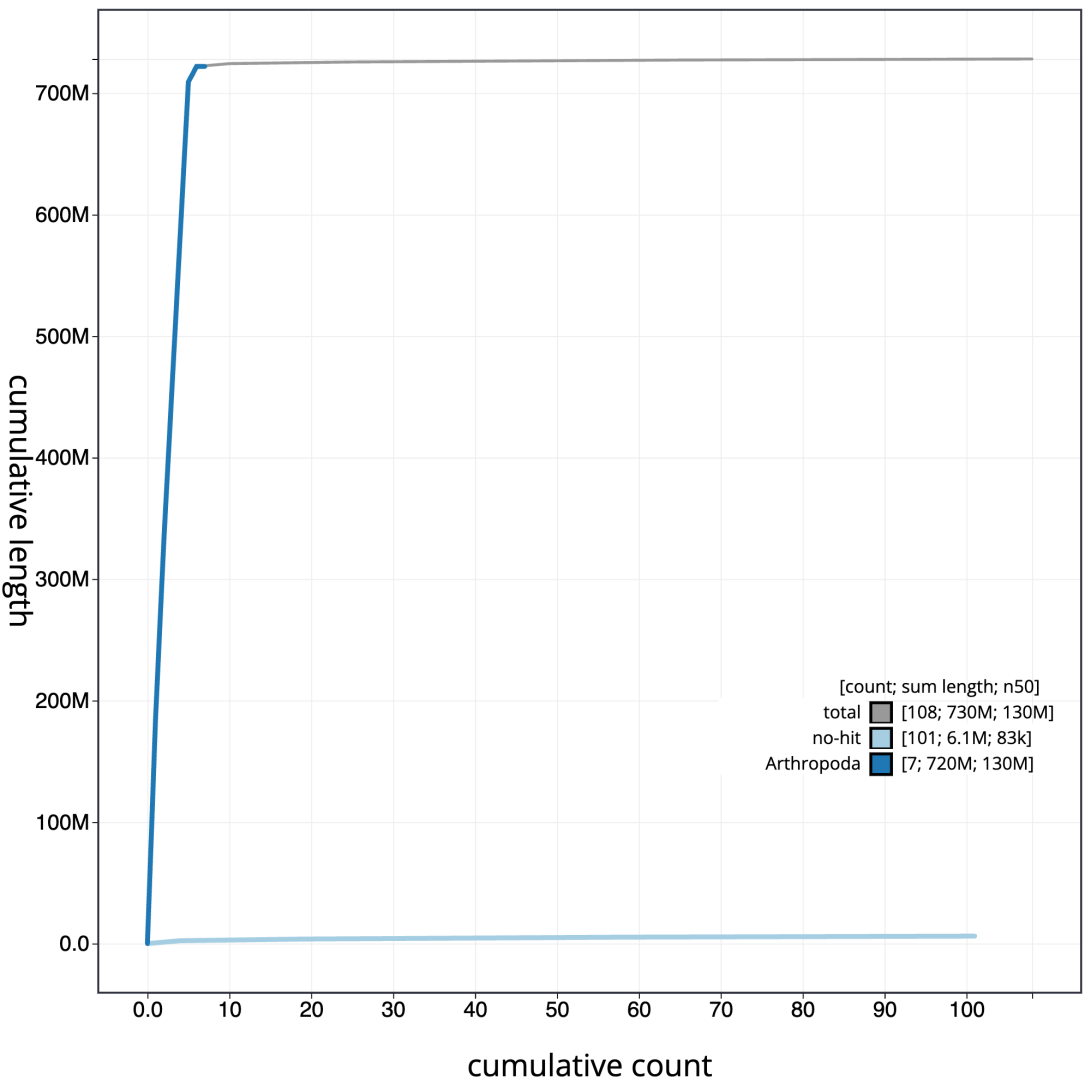
Genome assembly of *Stomorhina lunata*, idStoLuna1.1: BlobToolKit cumulative sequence plot. The grey line shows cumulative length for all scaffolds. Coloured lines show cumulative lengths of scaffolds assigned to each phylum using the buscogenes taxrule. An interactive version of this figure is available at https://blobtoolkit.genomehubs.org/view/idStoLuna1.1/dataset/CAKOFX01/cumulative.

**Figure 5.**
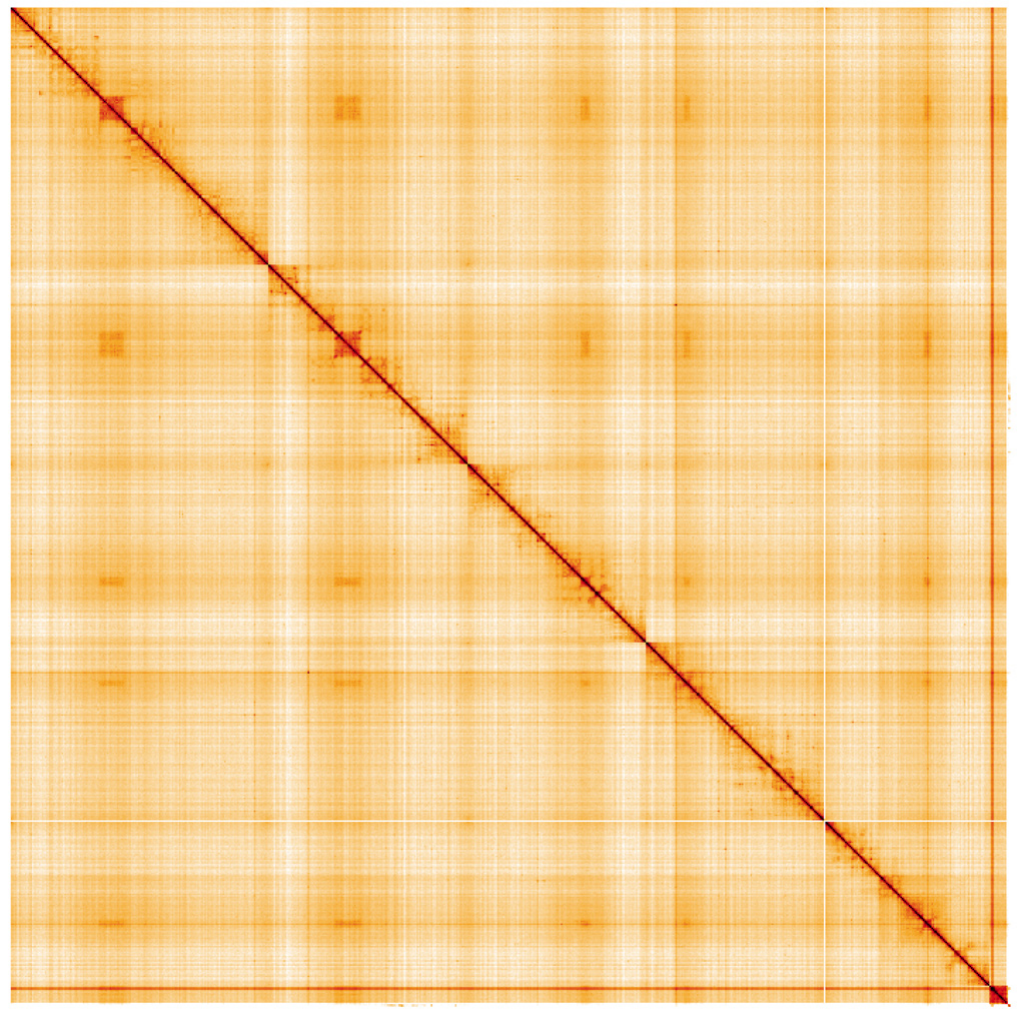
Genome assembly of *Stomorhina lunata*, idStoLuna1.1: Hi-C contact map of the idStoLuna1.1 alternate haplotype assembly, visualised using HiGlass. Chromosomes are shown in order of size from left to right and top to bottom. An interactive version of this figure may be viewed at https://genome-note-higlass.tol.sanger.ac.uk/l/?d=LbSE7mKdTZaujqShK85QNA.

**Table 2.** Chromosomal pseudomolecules in the genome assembly of *Stomorhina lunata*, idStoLuna1.

INSDC accession	Chromosome	Length (Mb)	GC%
OW121740.1	1	186.7	28.5
OW121741.1	2	144.44	29.0
OW121742.1	3	129.65	28.0
OW121743.1	4	129.06	27.5
OW121744.1	5	119.36	28.0
OW121745.1	X	12.71	31.5
OW121746.1	MT	0.02	21.0

The estimated Quality Value (QV) of the final assembly is 58 with *k*-mer completeness of 99.99%, and the assembly has a BUSCO v5.3.2 completeness of 99.3% (single = 98.8%, duplicated = 0.5%), using the diptera_odb10 reference set ( *n* = 3,285).

Metadata for specimens, spectral estimates, sequencing runs, contaminants and pre-curation assembly statistics can be found at https://links.tol.sanger.ac.uk/species/1606781.

## Genome annotation report

The *Stomorhina lunata* genome assembly (GCA_933228675.1) was annotated using the Ensembl rapid annotation pipeline ( Table 1; https://rapid.ensembl.org/Stomorhina_lunata_GCA_933228675.1/Info/Index). The resulting annotation includes 18,358 transcribed mRNAs from 11,857 protein-coding and 1,492 non-coding genes.

## Methods

### Sample acquisition and nucleic acid extraction

A female *Stomorhina lunata* (idStoLuna1) was collected from Hartslock Reserve, Oxfordshire, UK (latitude 51.51, longitude –1.11) on 2020-08-20. The specimen was collected by Ryan Mitchell (Natural History Museum) using an aerial net. The specimen was identified by the collector and snap-frozen on dry ice. 

DNA was extracted at the Tree of Life laboratory, Wellcome Sanger Institute (WSI). The idStoLuna1 sample was weighed and dissected on dry ice with tissue set aside for Hi-C sequencing. Thorax tissue was disrupted using a Nippi Powermasher fitted with a BioMasher pestle. High molecular weight (HMW) DNA was extracted using the Qiagen MagAttract HMW DNA extraction kit. HMW DNA was sheared into an average fragment size of 12–20 kb in a Megaruptor 3 system with speed setting 30. Sheared DNA was purified by solid-phase reversible immobilisation using AMPure PB beads with a 1.8X ratio of beads to sample to remove the shorter fragments and concentrate the DNA sample. The concentration of the sheared and purified DNA was assessed using a Nanodrop spectrophotometer and Qubit Fluorometer and Qubit dsDNA High Sensitivity Assay kit. Fragment size distribution was evaluated by running the sample on the FemtoPulse system.

RNA was extracted from abdomen tissue of idStoLuna1 in the Tree of Life Laboratory at the WSI using TRIzol, according to the manufacturer’s instructions. RNA was then eluted in 50 μl RNAse-free water and its concentration assessed using a Nanodrop spectrophotometer and Qubit Fluorometer using the Qubit RNA Broad-Range (BR) Assay kit. Analysis of the integrity of the RNA was done using Agilent RNA 6000 Pico Kit and Eukaryotic Total RNA assay.

### Sequencing

Pacific Biosciences HiFi circular consensus DNA sequencing libraries were constructed according to the manufacturers’ instructions. Poly(A) RNA-Seq libraries were constructed using the NEB Ultra II RNA Library Prep kit. DNA and RNA sequencing was performed by the Scientific Operations core at the WSI on Pacific Biosciences SEQUEL II (HiFi) and Illumina NovaSeq 6000 (RNA-Seq) instruments. Hi-C data were also generated from head tissue of idStoLuna1 using the Arima2 kit and sequenced on the Illumina NovaSeq 6000 instrument.

### Genome assembly, curation and evaluation

Assembly was carried out with Hifiasm ( Cheng *et al.*, 2021) and haplotypic duplication was identified and removed with purge_dups ( Guan *et al.*, 2020). The assembly was then scaffolded with Hi-C data ( Rao *et al.*, 2014) using YaHS ( Zhou *et al.*, 2023). The assembly was checked for contamination and corrected using the gEVAL system ( Chow *et al.*, 2016) as described previously ( Howe *et al.*, 2021). Manual curation was performed using gEVAL, HiGlass ( Kerpedjiev *et al.*, 2018) and Pretext ( Harry, 2022). The mitochondrial genome was assembled using MitoHiFi ( Uliano-Silva *et al.*, 2022), which runs MitoFinder ( Allio *et al.*, 2020) or MITOS ( Bernt *et al.*, 2013) and uses these annotations to select the final mitochondrial contig and to ensure the general quality of the sequence.

A Hi-C map for the final assembly was produced using bwa-mem2 ( Vasimuddin *et al.*, 2019) in the Cooler file format ( Abdennur & Mirny, 2020). To assess the assembly metrics, the *k*-mer completeness and QV consensus quality values were calculated in Merqury ( Rhie *et al.*, 2020). This work was done using Nextflow ( Di Tommaso *et al.*, 2017) DSL2 pipelines “sanger-tol/readmapping” ( Surana *et al.*, 2023a) and “sanger-tol/genomenote” ( Surana *et al.*, 2023b). The genome was analysed within the BlobToolKit environment (Challis *et al.*, 2020) and BUSCO scores ( Simão *et al.*, 2015; Manni *et al.*, 2021) were calculated.

Table 3 contains a list of relevant software tool versions and sources.

**Table 3.** Software tools: versions and sources.

Software tool	Version	Source
BlobToolKit	3.4.0	https://github.com/blobtoolkit/blobtoolkit
BUSCO	5.3.2	https://gitlab.com/ezlab/busco
gEVAL	N/A	https://geval.org.uk/
Hifiasm	0.16.1-r375	https://github.com/chhylp123/hifiasm
HiGlass	1.11.6	https://github.com/higlass/higlass
Merqury	MerquryFK	https://github.com/thegenemyers/MERQURY.FK
MitoHiFi	2	https://github.com/marcelauliano/MitoHiFi
PretextView	0.2	https://github.com/wtsi-hpag/PretextView
purge_dups	1.2.3	https://github.com/dfguan/purge_dups
sanger-tol/genomenote	v1.0	https://github.com/sanger-tol/genomenote
sanger-tol/readmapping	1.1.0	https://github.com/sanger-tol/readmapping/tree/1.1.0
YaHS	yahs-1.1.91eebc2	https://github.com/c-zhou/yahs

### Genome annotation

The Ensembl gene annotation system ( Aken *et al.*, 2016) was used to generate annotation for the *Stomorhina lunata* assembly (GCA_933228675.1). Annotation was created primarily through alignment of transcriptomic data to the genome, with gap filling via protein-to-genome alignments of a select set of proteins from UniProt ( UniProt Consortium, 2019).

### Legal and ethical review process for Darwin Tree of Life Partner submitted materials

The materials that have contributed to this genome note have been supplied by a Darwin Tree of Life Partner.

The submission of materials by a Darwin Tree of Life Partner is subject to the **‘Darwin Tree of Life Project Sampling Code of Practice’**, which can be found in full on the Darwin Tree of Life website here. By agreeing with and signing up to the Sampling Code of Practice, the Darwin Tree of Life Partner agrees they will meet the legal and ethical requirements and standards set out within this document in respect of all samples acquired for, and supplied to, the Darwin Tree of Life Project.

Further, the Wellcome Sanger Institute employs a process whereby due diligence is carried out proportionate to the nature of the materials themselves, and the circumstances under which they have been/are to be collected and provided for use. The purpose of this is to address and mitigate any potential legal and/or ethical implications of receipt and use of the materials as part of the research project, and to ensure that in doing so we align with best practice wherever possible.

The overarching areas of consideration are:

- Ethical review of provenance and sourcing of the material
- Legality of collection, transfer and use (national and international)

Each transfer of samples is further undertaken according to a Research Collaboration Agreement or Material Transfer Agreement entered into by the Darwin Tree of Life Partner, Genome Research Limited (operating as the Wellcome Sanger Institute), and in some circumstances other Darwin Tree of Life collaborators.

## Data Availability

European Nucleotide Archive: *Stomorhina lunata*. Accession number PRJEB50881; https://identifiers.org/ena.embl/PRJEB50881. ( Wellcome Sanger Institute, 2022) The genome sequence is released openly for reuse. The *Stomorhina lunata* genome sequencing initiative is part of the Darwin Tree of Life (DToL) project. All raw sequence data and the assembly have been deposited in INSDC databases. Raw data and assembly accession identifiers are reported in Table 1.

## Author information

Members of the Natural History Museum Genome Acquisition Lab are listed here:

https://doi.org/10.5281/zenodo.4790042.

Members of the Darwin Tree of Life Barcoding collective are listed here: https://doi.org/10.5281/zenodo.4893703.

Members of the Wellcome Sanger Institute Tree of Life programme are listed here: https://doi.org/10.5281/zenodo.4783585.

Members of Wellcome Sanger Institute Scientific Operations: DNA Pipelines collective are listed here: https://doi.org/10.5281/zenodo.4790455.

Members of the Tree of Life Core Informatics collective are listed here: https://doi.org/10.5281/zenodo.5013541.

Members of the Darwin Tree of Life Consortium are listed here: https://doi.org/10.5281/zenodo.4783558.
