# Supplementary material for: The genome sequence of the Locust Fly, Stomorhina lunata (Fabricius, 1805)
Source: Wellcome Open Res. 2023 Aug 2;8:330. [Version 1] doi: 10.12688/wellcomeopenres.19532.1 (PMC11109694; doi:10.12688/wellcomeopenres.19532.1)
Supplement: Peer review history

# Reviewer report: Reviewer response for version 1

**DOI:** 10.21956/wellcomeopenres.21636.r82000

**Published:** 2024-05-21

**Authors:** Peter Arensburger

**Affiliations:** 1 California State Polytechnic University Pomona, Pomona, California, USA

The authors of “The genome sequence of the Locust Fly, *Stomorhina lunata *(Fabricius, 1805)” present a thoroughly researched data note regarding the sequencing of this fly. They provide relevant historical context of previous work on this species as well as comprehensive descriptions of collection, sequencing, and data analysis methods. I cannot find any fault with this work.

Are sufficient details of methods and materials provided to allow replication by others?

Yes

Is the rationale for creating the dataset(s) clearly described?

Yes

Are the datasets clearly presented in a useable and accessible format?

Yes

Are the protocols appropriate and is the work technically sound?

Yes

Reviewer Expertise:

Arthropod molecular biology, bioinformatics, transposable elements

I confirm that I have read this submission and believe that I have an appropriate level of expertise to confirm that it is of an acceptable scientific standard.

## References

[1] : Merodon aureus Fabricius, 1805. *Fabricius, J.C. 1805. Systema antliatorum secundum ordines, genera, species... xiv + 15-372 + [1] + 30 pp. C. Reichard, Brunsvigae [* .1805;

# Reviewer report: Reviewer response for version 1

**DOI:** 10.21956/wellcomeopenres.21636.r64547

**Published:** 2023-08-21

**Authors:** Le Kang

**Affiliations:** 1 Institute of Zoology, Chinese Academy of Sciences, Beijing, 100101, China

The paper showcases the genome assembly and annotation of Stomorhina lunata, commonly known as the Locust Fly. The genome spans 728.1 megabases and is organized into 6 chromosomal pseudomolecules, encompassing the X sex chromosome. The mitochondrial genome, covering 16.49 kilobases, has also been successfully assembled. Ensembl-based gene annotation unveiled a total of 18,358 protein-coding genes. The sequencing employed Pacific Biosciences single-molecule HiFi long reads, followed by assembly refinement through curation efforts. The genome annotation process involved aligning transcriptomic data and specific UniProt proteins to the genome. This study significantly contributes to the Darwin Tree of Life Project, which aims to sequence all eukaryotic species in the Atlantic Archipelago of Britain and Ireland. The assembled genome and annotations serve as invaluable sources for advancing our further understanding of this species and its ecological role. A minor point of contention pertains to the lack of a detailed explanation regarding identifying the X sex chromosome.

Are sufficient details of methods and materials provided to allow replication by others?

Partly

Is the rationale for creating the dataset(s) clearly described?

Yes

Are the datasets clearly presented in a useable and accessible format?

Yes

Are the protocols appropriate and is the work technically sound?

Yes

Reviewer Expertise:

Insect genomics

I confirm that I have read this submission and believe that I have an appropriate level of expertise to confirm that it is of an acceptable scientific standard.
